# Fast‐Response Oxygen Optical Fiber Sensor based on PEA_2_SnI_4_ Perovskite with Extremely Low Limit of Detection

**DOI:** 10.1002/advs.202104708

**Published:** 2022-01-17

**Authors:** Shunshuo Cai, Yangyang Ju, Yangming Wang, Xiaowei Li, Tuan Guo, Haizheng Zhong, Lingling Huang

**Affiliations:** ^1^ Beijing Engineering Research Center of Mixed Reality and Advanced Display School of Optics and Photonics Beijing Institute of Technology Beijing 100081 China; ^2^ MIIT Key Laboratory for Low‐dimensional Quantum Structure and Devices School of Materials Science & Engineering Beijing Institute of Technology Beijing 100081 China; ^3^ Laser Micro/Nano‐Fabrication Laboratory School of Mechanical Engineering Beijing Institute of Technology Beijing 100081 China; ^4^ Institute of Photonics Technology Jinan University Guangzhou 510632 China

**Keywords:** optical fiber sensor, oxygen sensor, tilted fiber Bragg grating, tin perovskites

## Abstract

Oxygen sensor is an important technique in various applications including industrial process control, medical equipment, biological fabrication, etc. The reported optical fiber‐based configurations so far, using gas‐sensitive coating do not meet the stringent performance targets, such as fast response time and low limit of detection (LOD). Tin‐based halide perovskites are sensitive to oxygen with potential use for sensor applications. Here, the halide perovskite‐based oxygen optical fiber sensor by combining phenylethylammonium tin iodide (PEA_2_SnI_4_) and tilted fiber Bragg grating (TFBG) is demonstrated. The PEA_2_SnI_4_‐based oxygen optical fiber sensor is reversible at room temperature with a response time of about 10 s, and the experimental LOD approaches to an extremely low oxygen concentration of about 50 ppm. The as‐fabricated oxygen sensor shows a relative response change of 0.6 dB for an oxygen concentration increase from 50 ppm to 5% with good gas selection against NO_2_, CO, CO_2_, H_2_. This work extends the sensor applications of halide perovskites, providing a novel technique for rapid and repeatable oxygen gas detection at a low level.

## Graphical abstract

In this work, a 2D Sn‐based perovskites‐coated tilted fiber Bragg grating oxygen sensor is proposed. The sensor shows an extremely low experimental limit of detection down to 50 ppm and a fast response time less than 10 s. The combination of halide perovskites and optical fiber opens up the possibility to develop various smart sensors for artificial intelligence technology.

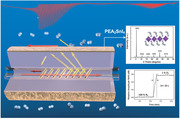

## 1 Introduction

Oxygen sensors play a pivotal role in many fields including industrial process control, medical equipment, and biological fabrication.^[^ ^1^ ^]^ Among the various applications, there is a special need to ensure the absence of oxygen or maintain at very low concentration, in particular for nitrogen (N_2_) generation, industrial gas manufacturing, anaerobic microorganisms growth monitoring, and food packaging.^[^ ^2^, ^3^ ^]^ Currently, the commercially available trace oxygen sensors are based on electrochemical, zirconia, and paramagnetic technologies.^[^ ^4^, ^5^, ^6^ ^]^ Electrochemical sensors are the most widely used low‐cost and accurate technique with a reasonable operational lifetime, however suffering from long recovery times and short reliable lifetime.^[^ ^7^, ^8^ ^]^ Although zirconia and paramagnetic‐based oxygen sensors can offer longer operational lifetimes, the high price of paramagnetic and high operation temperature of zirconia limit their wide utilization. Therefore, it has been an increased demand to develop low‐cost and fast‐response trace oxygen sensors with ppm detection level at room temperature.

Optical fiber sensor based on the change of optical properties with response to the environmental parameters have received a great number of attentions and successfully applied in many fields.^[^ ^9^, ^10^, ^11^ ^]^ It has been well known that the interactions of oxygen with materials can induce the variation of absorption spectra, photoluminescence (PL) properties, reflective index, Raman spectra, surface plasma resonance, etc.^[^ ^12^, ^13^, ^14^ ^]^ By selecting oxygen sensitive materials, it has been of great interest to develop oxygen optical fiber sensor. Up to date, most of the reported oxygen optical fiber sensors are based on the luminescence quenching of sensitive materials on optical fiber tips.^[^ ^15^, ^16^, ^17^ ^]^ However, the luminescence‐based optical fiber sensors show drawbacks such as stringent requirements for excitation light sources (wavelength, excitation power) and self‐quenching of oxygen sensing material. Therefore, it is very necessary to develop alternative oxygen optical fiber sensors.

Tilted fiber Bragg grating (TFBG) is an emerging optical fiber technique to break the cylindrical symmetry with tilted grating structure.^[^ ^18^, ^19^, ^20^ ^]^ The tilted grating structure induced strong evanescent field, which can respond to the refractive index change at an extreme low limit of detection (LOD). Considering the crucial factor of refractive index in TFBG‐based sensors, TFBG provides an alternative parameter of sensing material to develop oxygen sensors.^[^ ^21^, ^22^ ^]^ Sn‐based lead‐free perovskites have been extensively investigated as low toxic solution processed semiconductors for solar cells^[^ ^23^, ^24^, ^25^ ^]^ as well as other optoelectronic applications.^[^ ^26^, ^27^ ^]^ Because of their high surface‐to‐volume ratio and the low inherently redox potential of the Sn(II)/Sn(IV) couple,^[^ ^28^ ^]^ Sn‐based perovskites could be a promising candidate as oxygen‐sensitive material for oxygen sensing.^[^ ^29^, ^30^, ^31^, ^32^, ^33^ ^]^ Phenylethylammonium tin iodide (PEA_2_SnI_4_) is a typical 2D‐layered Sn‐based perovskites with moderate stability against heating, light, and moisture.^[^ ^34^, ^35^ ^]^ In the present work, we demonstrate the first reversible halide perovskite‐based oxygen optical fiber sensor by combining PEA_2_SnI_4_ and TFBG. The optical response of 2D‐layered PEA_2_SnI_4_ to low oxygen level is studied and a LOD of about 50 ppm was achieved.

## 2 Results

**Figure 1:**
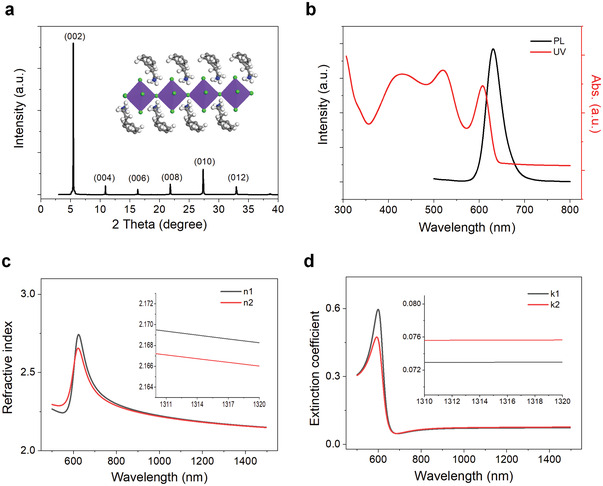
Crystal structure and optical properties of PEA_2_SnI_4_ perovskite. a) X‐ray diffraction (XRD) spectra and crystal schematic structure of fresh PEA_2_SnI_4_ film. b) UV‐vis and PL spectra of PEA_2_SnI_4_ perovskite. c) Refractive index (*n*) and d) extinction coefficient (*k*) of PEA_2_SnI_4_ polycrystalline thin film in a nitrogen atmosphere (black curve) and upon oxygen exposure (50% [O_2_], red curve) using ellipsometry (VB250, VASE, J. A. Woollam, USA).

PEA_2_SnI_4_ was fabricated by dip coating of a mixed precursor solution of PEAI and SnI_2_ (molar ratio of 2:1) in mixed solvents of *N*,*N*‐dimethylformamide and dimethyl sulfoxide (volume ratio of 4:1). The crystal structure of the as‐fabricated perovskites was identified by applying X‐ray diffraction (XRD) characterization. As shown in **Figure** **1**a, the XRD patterns show typical diffraction peaks of 2D‐layered perovskites with peaks corresponding to (0 0 *l*) planes (*l* = 2, 4, 6, 8, and 12). The resulting PEA_2_SnI_4_ thin film was further investigated using UV‐vis absorption (UV‐vis) and photoluminescence (PL) spectra. As shown in Figure 1b, the observed three main absorption peaks, that located at 430 nm (2.88 eV), 521 nm (2.38 eV), and 607 nm (2.04 eV), can be assigned to the high energy exciton transition energy levels,^[^ ^36^ ^]^ the charge transfer transition between the organic spacer cations and inorganic layers,^[^ ^37^ ^]^ and the intrinsic exciton absorption,^[^ ^38^ ^]^ respectively. The resulting PEA_2_SnI_4_ thin film shows narrow‐band red PL emission with peak located at 633 nm (1.97 eV). As can be seen in Figure 1c,d, the refractive index of as‐fabricated PEA_2_SnI_4_ thin film under oxygen exposure was investigated using ellipsometry. The detail of data analysis is described in the Supporting Information. As summarized in Table S1 in the Supporting Information, the refractive index (*n*) and extinction coefficient (*k*) changed when the sample is exposed to oxygen. It is obvious that the refractive index of PEA_2_SnI_4_ thin film can be greatly varied upon oxygen exposure.

A layer of PEA_2_SnI_4_ thin film was then deposited on the surface of the SiO_2_‐based 125 µm diameter fiber using the spin‐coating technique. Figure S1 in the Supporting Information shows the scanning electron microscope (SEM, Thermo DXR2) image of the surface of a bare TFBG overcoated with a PEA_2_SnI_4_ film. It shows that the as‐fabricated PEA_2_SnI_4_ film on TFBG has a uniform surface with a thickness of about 500 nm.

**Figure 2:**
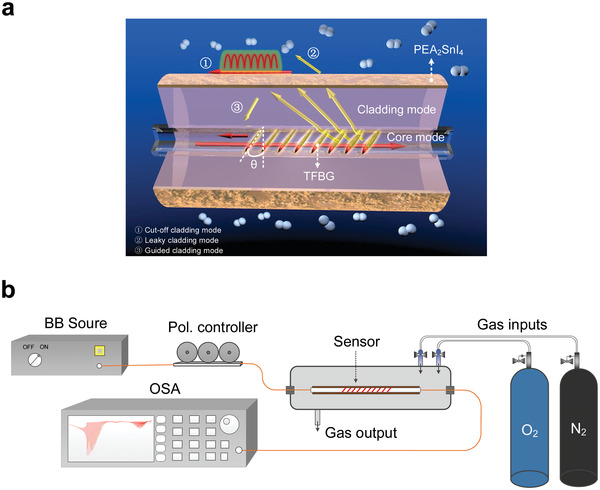
a) The schematic of the TFBG in terms of cut‐off surface resonance oxygen sensing principle. b) The setup to clarify the sensing characteristics of the oxygen environment.

**Figure** **2**a schematically shows the structure of constructed oxygen optical fiber sensor. A 16 mm long TFBG in a commercial telecom single‐mode fiber core was fabricated using the phase‐mask technique and then used as a platform to construct the optical fiber sensor. As shown in Figure 2a, TFBG can couple the incident core mode to generate reflected core mode as well as three reflected cladding modes including guided cladding modes, cut‐off mode, and leaky cladding modes.

The guided cladding modes are totally internally reflection inside the fiber cladding due to the higher effective refractive index (ERI) of cladding modes than the surrounding refractive index (SRI). When ERI is smaller than the SRI, leaky cladding modes appear.^[^ ^39^ ^]^ The cut‐off mode is an evanescent field in fiber cladding, when ERI approaches to SRI. It has been learned that the cut‐off mode is extremely sensitive to SRI. Because SRI can be potentially varied with the parameter change of environmental condition, the cut‐off mode of TFBG has been widely applied in constructing fiber sensors.^[^ ^40^ ^]^

The tilt angle (*θ*) between incident laser beam and fiber normal direction was controlled to tune cut‐off mode during the fabrication of TFBG. As a result, it is possible to adjust the operating wavelength of cut‐off mode for achieving fast response and high sensitivity to certain refractive index.^[^ ^41^ ^]^ In this work, the tilt angle of TFBG was set at 37^o^ to enhance the cut‐off mode amplitudes with a surrounding refractive index in the range of 0.9–1.1. As shown in Figure S2 in the Supporting Information, the cladding modes range from 1250 to 1560 nm and can be divided into two main subsets. The cladding modes in the wavelength between 1475 and 1550 nm have an effective refractive index of 1.33–1.45. Consequently, this group is suitable to measure the targets in aqueous solutions. The cladding modes in the wavelength between 1250 and 1400 nm are sensitive to the targets with effective refractive index of 0.90–1.1, providing a platform for gas sensor applications.^[^ ^42^, ^43^ ^]^

The performance of PEA_2_SnI_4_‐based oxygen optical fiber sensor was then investigated by incorporating the PEA_2_SnI_4_‐coated fiber into a home‐made set up. As illustrated in Figure 2b,  a broadband light source (BBS), working range from 1250 to 1600 nm, was connected to a polarization controller to ensure the input is linearly polarized for radial (*P*) polarization relative to the grating plane and maximize the coupling of energy to cut‐off mode resonance. Finally, the output spectrum was recorded by an optical spectrum analyzer (OSA, Yokogawa AQ6370D). Two gas inputs, i.e., oxygen (O_2_) and nitrogen (N_2_) are incorporated into the gas chamber. Hence, the O_2_ concentrations can be varied by adjusting the flow difference between the two gases.

**Figure 3:**
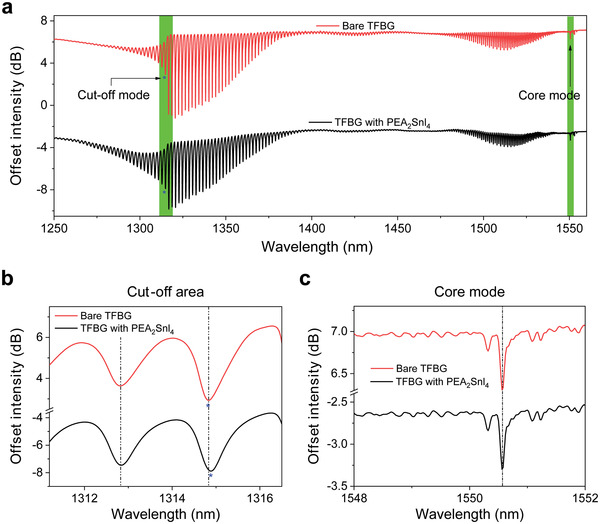
a) The transmitted amplitude spectra of a bare TFBG and PEA_2_SnI_4_‐coated TFBG in air; b) the enlarged detail of the cut‐off surface mode resonance; c) the core mode used as for temperature elimination.

The available refractive index change from PEA_2_SnI_4_‐integrated TFBG device provides a platform for designing optical oxygen sensors. We then track the cut‐off mode of PEA_2_SnI_4_‐coated TFBG for detecting the trace oxygen. **Figure** **3**a and Figure S3 in the Supporting Information show the full transmission spectrum of the PEA_2_SnI_4_‐integrated TFBG with a bare TFBG as comparison. The cut‐off mode is pointed out with a blue asterisk and is highlighted in green background. Figure 3b illustrates the intensity change and wavelength shift of the cut‐off mode of TFBG with PEA_2_SnI_4_ coating and the bare TFBG one. As shown in Figure 3c, the core mode of TFBG with PEA_2_SnI_4_ coating and the bare one is located at 1550.6 nm, the core mode is insensitive to the variations of surrounding refractive index. Thus, it can be used as a temperature reference when carrying out a spectrometer‐based interrogation.

**Figure 4:**
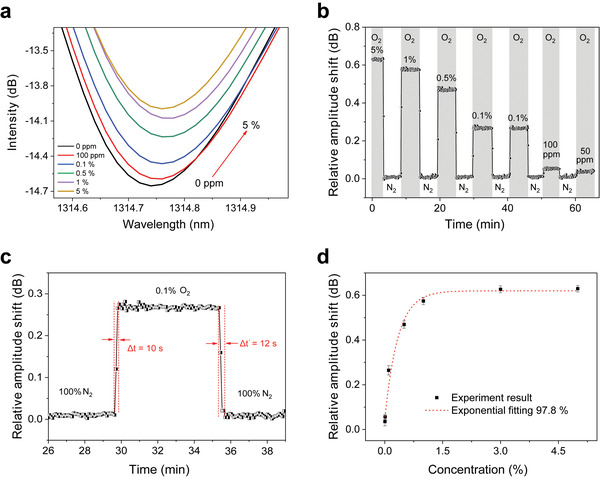
a) The cut‐off surface mode resonance when a sensor is exposed to oxygen with different concentration ranging from 50 ppm to 5%. b) The response to oxygen at a fixed concentration ranging from 0% to 5%. c) The sensors’ response and recovery time for oxygen detection at the concentration of 0.1%. d) The exponential response of the sensor with the concentration range from 50 ppm to 5%. The error bar represents the standard deviation of three independent measurements.

Under the exposure to oxygen with different concentrations, PEA_2_SnI_4_‐coated fiber surface exhibited a refractive index change, thus induced the spectra shift of cut‐off mode. As shown in **Figure** **4**a, the cut‐off mode shows gradual red shift with intensity increase with the refractive index change of the PEA_2_SnI_4_, when the oxygen concentration increases from 0 ppm to 0.5%. It is noted that a tiny blue‐shift is observed with the increasing concentration of 1% to 5% ppm. This can be explained to the saturation of PEA_2_SnI_4_ at higher oxygen concentration. Because the intensity change is more pronounced than the wavelength shift, we here tried to monitor the oxygen concentration using the intensity change.

Figure 4b presents the response curves of the PEA_2_SnI_4_‐coated TFBG sensor under different oxygen volumetric concentration (50 ppm, 100 ppm, 0.1%, 0.5%, 1%, and 5%). As it can be seen, the PEA_2_SnI_4_‐coated TFBG sensor can be recovered to the same baseline under a broad oxygen concentrations ranging from 50 ppm to 5%. An oxygen concentration of 50 ppm can be detected, corresponding to a relative intensity change of 0.0358 dB. As shown in Figure S4 in the Supporting Information, the PEA_2_SnI_4_‐coated TFBG sensor shows good reproducibility upon three cycles for the measurements at oxygen concentrations of 5%, 1%, 0.5%, 0.1%, 100 ppm, and 50 ppm, respectively. The standard deviation (*σ*, measurement noise) of the cut‐off mode amplitude was estimated to 0.0074 dB (see Figure S5, Supporting Information). Therefore, the theoretical LOD can be estimated in the user manner by using the experimental value equal to three times the standard deviation,^[^ ^44^, ^45^ ^]^ which corresponds to a concentration of 31 ppm.

Figure 4c presents the average stabilization time of the sensor during the oxygen‐induced lattice distortion phase. A rapid response time of ≈10 s at an oxygen concentration of 0.1% with a recovery time of ≈12 s was achieved for the PEA_2_SnI_4_‐coated TFBG oxygen sensor. As shown in Figure S6 in the Supporting Information, the response time varied from 10 s for 1% of oxygen to roughly 40 s at lower oxygen presence. The exponential response with a correlation coefficient of 97.8% is obtained at oxygen concentrations in nitrogen from 0% to 5% (see Figure 4d).

## 3 Discussion

**Figure 5:**
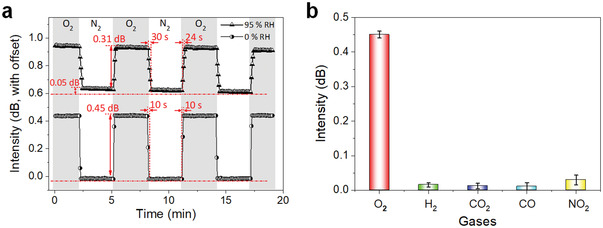
a) The response of PEA_2_SnI_4_‐coated TFBG sensor to oxygen in wet (top) and dry (bottom) atmosphere with the intensity and stabilization time values for association/recover phase. b) The responsibility to oxygen and other commonly used gases including hydrogen, carbon dioxide, carbon monoxide, and nitrogen dioxide with the concentration of 0.5%.

To illustrate the potential use of PEA_2_SnI_4_‐coated TFBG sensor for real applications, we here explored the gas selection against other gases. **Figure** **5**a presents the response of PEA_2_SnI_4_‐coated TFBG oxygen sensor at an oxygen concentration of 0.5% in the humid conditions with a relative humidity of 95%. Although the intensity changes were decreased from 0.45 to 0.31 dB, PEA_2_SnI_4_‐coated TFBG sensor still shows fast response and good reproducibility. The average stabilization time is about 24 s and the recovery phase is about 30 s. The gas selectivity of the PEA_2_SnI_4_‐coated TFBG sensor was also investigated. Figure 5b shows the selectivity test results upon exposure to four common gases including hydrogen, carbon dioxide, carbon monoxide, and nitrogen dioxide at the concentration of 0.5%. The intensity change of PEA_2_SnI_4_‐coated TFBG sensor is 0.015, 0.012, 0.01, and 0.29 dB for H_2_, CO_2_, CO, and NO_2_, respectively, which is an order lower than that of the response to oxygen. The good selectivity PEA_2_SnI_4_‐coated TFBG sensor can be correlated with the easy surface oxidation of Sn^2+^ to Sn^4+^, which has been demonstrated.^[^ ^25^, ^46^ ^]^

In summary, we here demonstrated the first 2D Sn‐based perovskites‐coated TFBG oxygen sensor. The sensor shows excellent sensitivity and fast response when exposing it to oxygen concentrations between 50 ppm and 5% in volume. Compared with the previously reported optical fiber oxygen sensors (see Table S2, Supporting Information), an extremely low experimental LOD down to 50 ppm and a fast response time less than 10 s corresponding to 1% oxygen concentration were achieved due to the high oxygen sensitivity of PEA_2_SnI_4_. Furthermore, the as‐fabricated oxygen sensor shows good responsivity in the humid condition as well as good gas selection against NO_2_, CO, CO_2_, H_2_. Together with the intrinsic features of optical fibers, these sensors provide a promising platform for oxygen detection where there is difficult access. Moreover, the combination of halide perovskites and optical fiber opens up the possibility to develop various smart sensors for artificial intelligence technology.

## 4 Experimental Section

### The Fabrication of TFBG

The TFBG sensor (16 mm in length) was fabricated using the phase‐mask technique in a commercial single‐mode fiber. The fabrication process mainly included the following three steps. First, the fiber was hydrogen‐loaded (temperature: 50 °C, pressure: 1500 psi, loading time: 168 h) to increase the photosensitivity of the fiber core. Second, pulsed 800 nm femtosecond light (power of 200 µJ per pulse, pulse duration of 125 fs) was focused with a cylinder lens and scanned over the fiber region. The phase mask divided the laser beam into ±1 order diffraction orders so as to induce a periodical effective refractive index modulation in the fiber core. Finally, by rotating the phase mask and the fiber simultaneously, a selectable tilt angle (relatively to the longitudinal axis of the fiber) could be easily induced into the grating.

### Optical Measurement

The PEA_2_SnI_4_‐coated TFBG oxygen‐sensing system employed is shown in Figure 2b, which comprised a broadband light source (Amonics ASLD‐CWDM‐5‐B‐FA, spectrum range: 1250–1650 nm), a polarization controller (PC), a gas chamber, an optical fiber sensing probe, and an optical spectrum analyzer (Yokogawa AQ6370D, detector range: 600–1700 nm, resolution: 0.02 nm). Two gas inputs, i.e., oxygen (O_2_) and nitrogen (N_2_) were incorporated into the gas chamber. Hence, the O_2_ concentrations could be varied by adjusting the flow difference between the two gases. The response to other gases was investigated by applying similar procedure.

## Conflict of Interest

The authors declare no conflict of interest.

## Author Contributions

S.C. and Y.J. contributed equally to this work. H.Z. and L.H. conceived and supervised the project. Y.J. and Y.W. fabricated the materials and conducted the material characterizations. S.C. and Y.J. carried out the spectroscopic measurements and the gas detection. T.G. and X.L. supported the fabrication of TFBG. S.C., Y.J.,H.Z., and L.H. wrote the paper with the discussion of other authors.

## Supporting information

Supporting Information

## Data Availability

Research data are not shared.
