# Supplementary material for: Fast‐Response Oxygen Optical Fiber Sensor based on PEA2SnI4 Perovskite with Extremely Low Limit of Detection
Source: Adv Sci (Weinh). 2022 Jan 17;9(8):2104708. doi: 10.1002/advs.202104708 (PMC8922120; doi:10.1002/advs.202104708)
Supplement: Supplementary file 1 — Supporting Information [file ADVS-9-2104708-s001.pdf]

ADVANCED SCIENCE
Open Access

Supporting Information

for *Adv. Sci.,* DOI: 10.1002/advs.202104708

Fast response oxygen optical fiber sensor based on PEA_2SnI_4 perovskite with extremely low limit of detection

Shunshuo Cai, Yangyang Ju, Yangming Wang, Xiaowei Li, Tuan Guo, Haizheng Zhong, and Lingling Huang**

Supporting Information

Fast response oxygen optical fiber sensor based on PEA_2SnI_4 perovskite with extremely low limit of detection

Shunshuo Cai[1#], Yangyang Ju[2#], Yangming Wang[2], Xiaowei Li[3], Tuan Guo[4], Haizheng Zhong[2], and Lingling Huang[1]**

Dr. S. Cai, and Prof. L. Huang
Beijing Engineering Research Center of Mixed Reality and Advanced Display, School of Optics and Photonics, Beijing Institute of Technology, 100081 Beijing, China
E-mail: (huanglingling@bit.edu.cn)

Dr. Y. Ju, Y. Wang, and Prof. H. Zhong
MIIT Key Laboratory for Low-dimensional Quantum Structure and Devices, School of Materials Science & Engineering, Beijing Institute of Technology, 100081 Beijing, China
E-mail: (hzzhong@bit.edu.cn)

Prof. X. Li
Laser Micro/Nano-Fabrication Laboratory, School of Mechanical Engineering, Beijing Institute of Technology, 100081 Beijing, China

Prof. T. Guo
Institute of Photonics Technology, Jinan University, 510632 Guangzhou, China

These authors contributed equally: Shunshuo Cai, Yangyang Ju

Data Analysis of Ellipsometry measuremetns.

To fit the ellipsometry measurement data, we use the Forouhi−Bloomer model to derive the refractive index upon oxygen exposure [1-3]. All the fitting parameters for PEA_2SnI_4 films upon oxygen exposure are listed in Table S1. The data analysis was achieved using the following equations from eq 1 to eq 5.

$$n(E) = n\,(\infty) + \sum_{i=1}^{m} \frac{B_{0i}E + C_{0i}}{E^2 - B_iE + C_i} \qquad \text{(eq 1)}$$

$$\text{k(E)} = \sum_{i=1}^{m} \frac{A_i(E-E_g)^2}{E^2 - B_iE + C_i} \qquad \text{(eq 2)}$$

$$B_{0i} = \frac{A_i}{Q_i}[-\frac{B_i^{\,2}}{2} + E_gB_i - E_g^{\,2} + C_i] \qquad \text{(eq 3)}$$

$$C_{0i} = \frac{A_i}{Q_i}[(E_g^{\,2} + C_i)\frac{B_i}{2} - 2E_gC_i] \qquad \text{(eq 4)}$$

$$Q_i = \frac{1}{2}(4C_i - B_i^{\,2})^{1/2} \qquad \text{(eq 5)}$$

where *n* and *k* are real and imaginary part of index of refraction, which are dispersive and depend on energy *E*. *E* is photon energy; E_g is the bandgap; A_i, B_i and C_i are constants, proportional to the square of the position matrix element (electron transition lifetime), twice the bandwidth difference between the conduction band and the valence band, and depends on the constants of A_i and B_i, respectively.

References

[1] Macleod H A. Thin-Film Optical Filters. *Bristol: Institute of Physics Publishing*, **2001**, 30-40.

[2] Forouhi A., Bloomer I., *Phys. Rev. B*, **1986**, 34, 7018.

[3] Liu B., Soe C. M. M., Stoumpos, C. C., Nie W., Tsai H., Lim K., Mohite A. D., Kanatzidis M. G., Marks T. J., Singer K. D., *Solar RRL*, **2017**, 1, 1700062.

Table. S1 All the fitting parameters for PEA_2SnI_4 films upon oxygen exposure.

Model Parameter	Pristine	The sample after oxygen exposure
Surface rough, nm	2.391	10.840
d, nm	165.461	133.241
n_∞	2.069	2.079
A_0	0.104	0.107
B_0	4.039	4.041
C_0	4.088	4.096
E_{g0}, eV	1.820	1.829
A_1	0.618	0.618
B_1	1.364	1.364
C_1	19.105	19.105
E_{g1}, eV	0.576	0.5761

Table. S2 Comparison between various optical fiber oxygen sensors reported in the literature and the one proposed in this work.

Time	Materials/ sensor type	Mechanism of sensor	Operation temperature	Response time	Recovery time	Limit of detection	Reference
2017/09	PAH/PAA/Pt / plastic silica cladding fiber sensor	quenching of fluorescence	no present	5-6 s @ 100 % O_2	11-14 s	no present	A. Nerea, *et al. Sensors and Actuators B*: *Chemical*, **2017**, 248, 836.
2015/02	PtTFPP complex / unclad plastic silica cladding fiber sensor	quenching of fluorescence	room temperature	>4 min. @ 100 % O_2	4 min.	no present	E. Cesar, et al. *Sensors and Actuators B: Chemical*, **2015**, 207, 683.
2018/03	PdTFPP / plastic optical fiber sensor	quenching of fluorescence	10–40 °C	10 s @ 100 % O_2	45 s	no present	Z. Maizatul, et al. *Optical Fiber Technology*, **2018**, 41, 109.
2014/05	PtTFPP / plastic optical fiber sensor	quenching of fluorescence	25–66 °C	6.7 s @ 100 % O_2	46.6 s	no present	C. Cheng-Shane, et al. *Sensors and Actuators B: Chemical*, **2014**, 195, 259.
2020/10	EuNDC / taped optical fiber sensor	quenching of fluorescence	room temperature	10 s @ 100 % O_2	70 s	210 ppm	X. Tifeng, et al. *Microporous and Mesoporous Materials*, **2020**, 305, 110396.
2017/09	Tb^{3+} @Bio-MOF-1	quenching of fluorescence	room temperature	no present	no present	490 ppm	W. Han, et al. *Journal of colloid and interface science*, **2017**, 502, 8.
2017/10	Nanostructured organo-metal halide perovskite / piezoelectricity sensor	current	room temperature	400 ms @ 5.5 % O_2	10 s	70 ppm	S. Marc-Antoine, et al. *Advanced Materials*, **2017**, 29, 1702469.
2017/07	Fe(II)-Polymer Wrapped Carbon Nanotubes/near-field communication (NFC) sensor	resistance	room temperature	50 s @ 2 %-21 % O_2	no present	0.3 %	Z. Rong, et al. *ACS sensors* **2017**, 2,1044-1050.
at present	PEA_2SnI_4 / tilted fiber Bragg grating sensor	refractive	room temperature	10 s @ 0.1 % O_2	10 s	50 ppm	**This work**

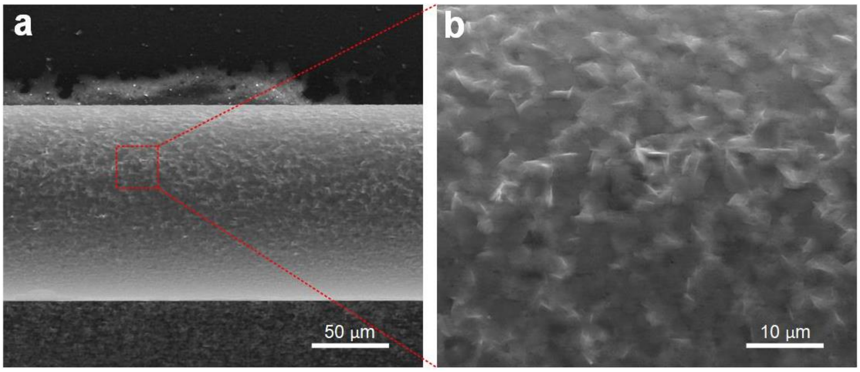

Figure. S1. The PEA_2SnI_4 film-coated TFBG using the spin-coating technology. a) SEM images showing the surface-section of PEA_2SnI_4 film-coated TFBG; b) enlarged view of the PEA_2SnI_4 film.

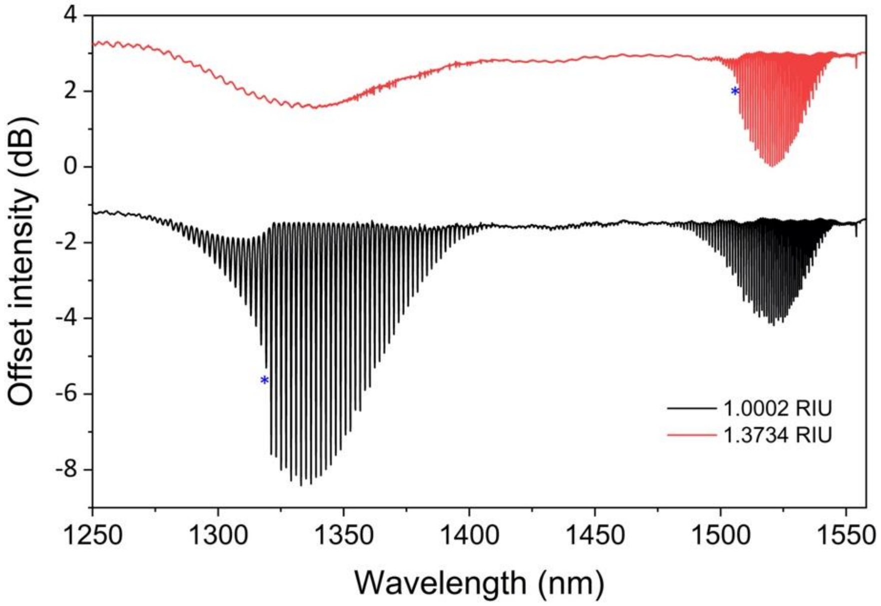

Figure. S2. Transmission spectra of bare 37° TFBG as a function of SRI (offset on the vertical scale, and the cut-off position is marked by the blue asterisk).

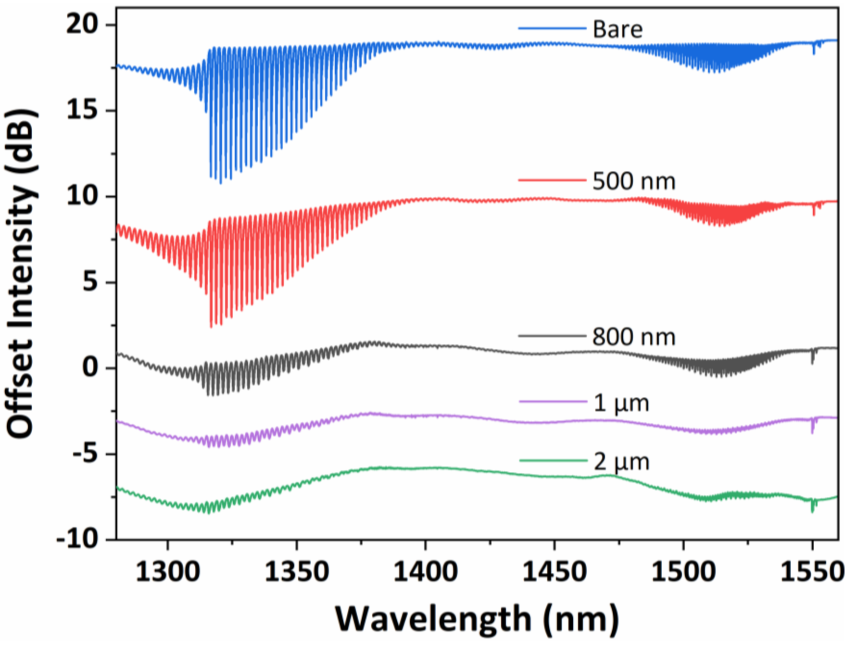

Figure. S3. The transmitted amplitude spectra of a bare TFBG and different thickness of PEA2SnI4 coated in the air.

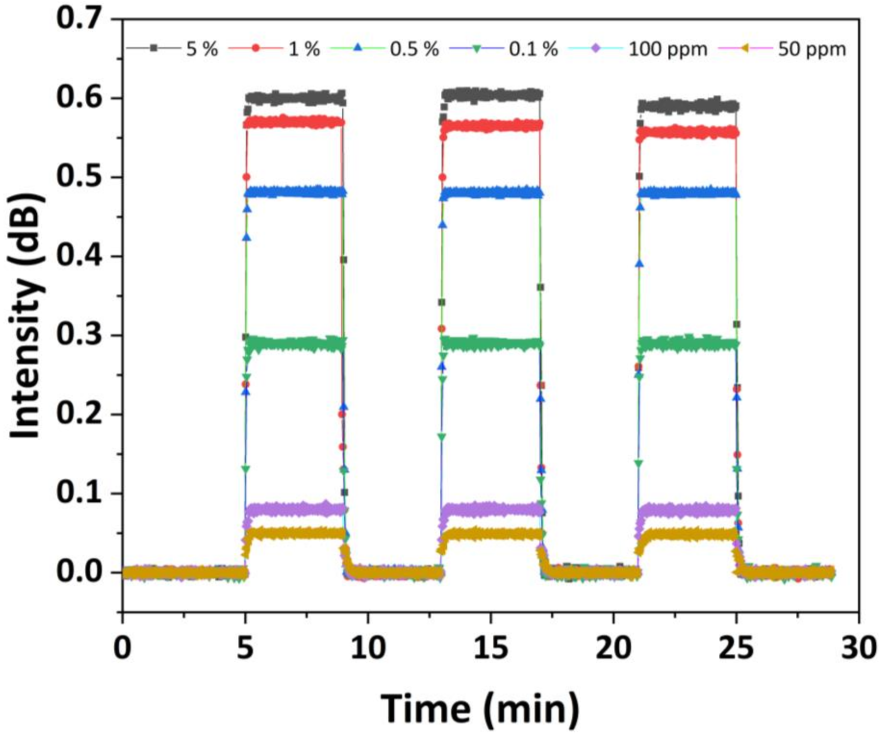

Figure. S4. Three cycles of the sensor's response to variation oxygen concentrations of 5%, 1%, 0.5%, 0.1%, 100 ppm, and 50 ppm.

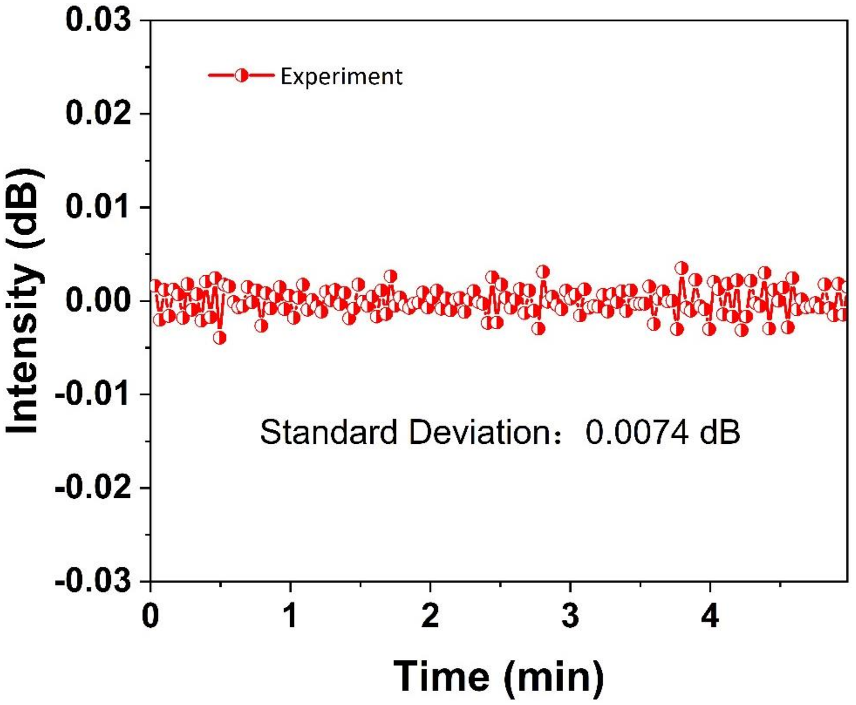

Figure. S5. Response of the cut-off mode amplitude to exposure of the sensor to a constant oxygen concentration of 0 %.

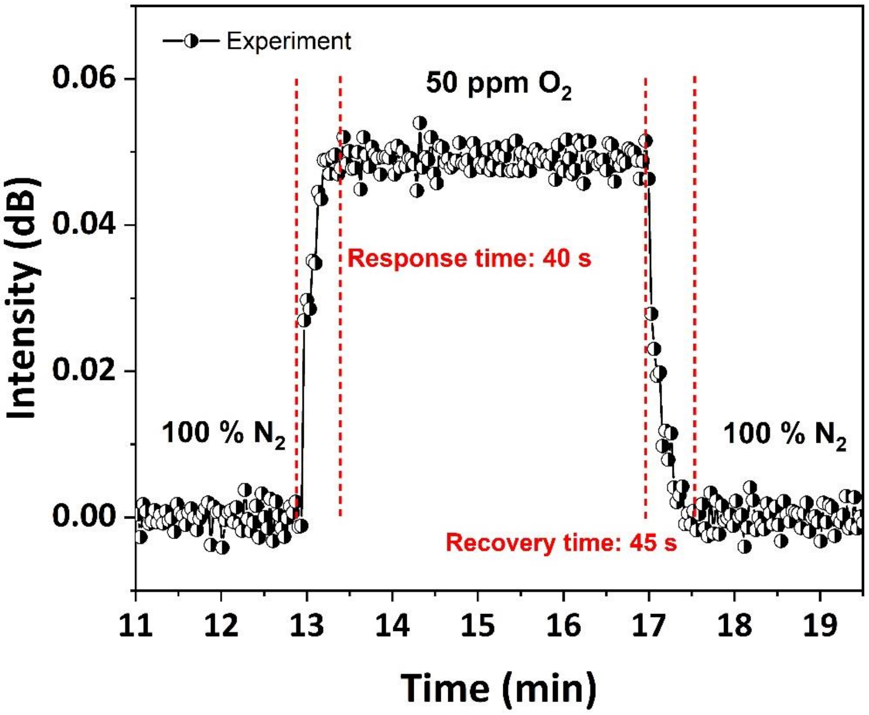

Figure. S6. Sensor's response time for oxygen detection with the concentration of 50 ppm in volume.
